# Scrotal Cystocele in a Sliding Left Inguinoscrotal Hernia: A Case Report and Review of Literature

**DOI:** 10.5005/jp-journals-l0018-1220

**Published:** 2017-05-05

**Authors:** Ambikavathy Mohan, Kumar Srinivasan

**Affiliations:** 1 Department of Surgery, Vydehi Institute of Medical Sciences and Research Centre, Bengaluru, Karnataka, India; 2 Department of General Medicine, Vydehi Institute of Medical Sciences & Research Centre, Bengaluru, Karnataka, India

**Keywords:** Bladder herniation, Scrotal cystocele, Sliding inguinoscrotal hernia.

## Abstract

Inguinoscrotal bladder herniation has a reported incidence of 1 to 4%. Although small bladder herniations are noted at the time of inguinal hernia repair, large bladder herniation into the scrotum is rare. These patients have a unique presentation of signs and symptoms. We report a case of a male patient of age 64 years who had a large inguinoscrotal hernia with bladder. He successfully underwent hernioplasty after repositioning of the bladder. Now, he is symptom-free and on follow-up.

**How to cite this article:** Mohan A, Srinivasan K. Scrotal Cystocele in a Sliding Left Inguinoscrotal Hernia: A Case Report and Review of Literature. Euroasian J Hepato-Gastroenterol 2017;7(1):87-88.

## INTRODUCTION

Inguinoscrotal hernias with bladder herniation are rare, with incidence of 1 to 4%.^1,2^ A preoperative diagnosis is very important to avoid bladder injury at the time of surgery. Literatures have cited only about 7% of cases to have been diagnosed before surgery.^2-4^ Ultrasonography, voiding cystourethrography, and magnetic resonance imaging (MRI) play an important role in preoperative diagnosis.^1,5-8^ We report a case of scrotal cystocele in a 64-year-old patient who underwent hernioplasty after bladder repositioning.

## CASE REPORT

An elderly male patient aged 64 years presented to surgical outpatient department with history of an intermittent swelling in the left groin for 6 to 8 months. He gave a typical symptom of increase in size of the swelling and double micturition (desire to void twice within a short time interval). By manual compression of the inguino-scrotal swelling, he noted decrease in the size of groin swelling following micturition. He did not have symptoms of thinning of stream, straining at micturition, or hesitancy. He was hypertensive and diabetic with regular treatment from the physician.

**Fig. 1:**
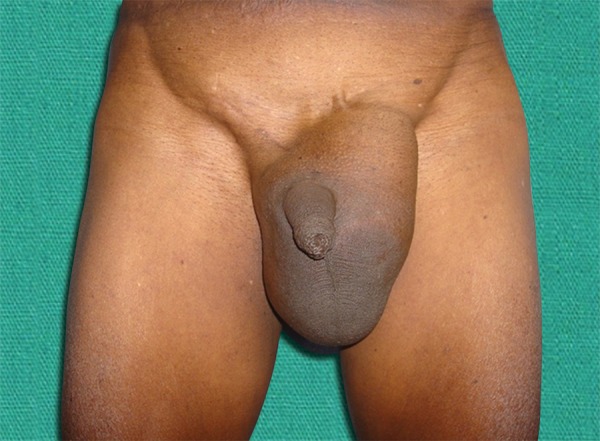
Preoperative view of left inguinoscrotal swelling -scrotal cystocele

**Fig. 2:**
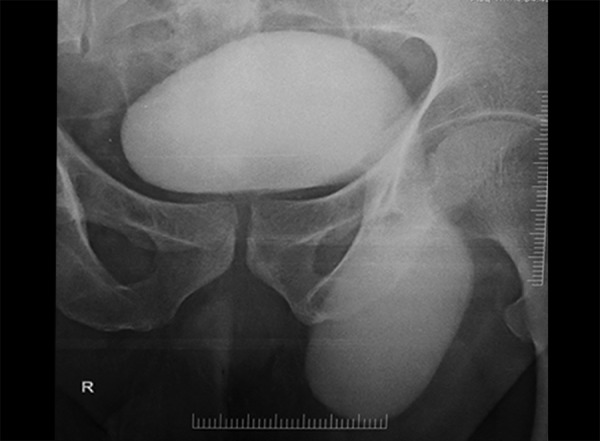
Voiding cystourethrography showing dog ear herniation of bladder

**Fig. 3:**
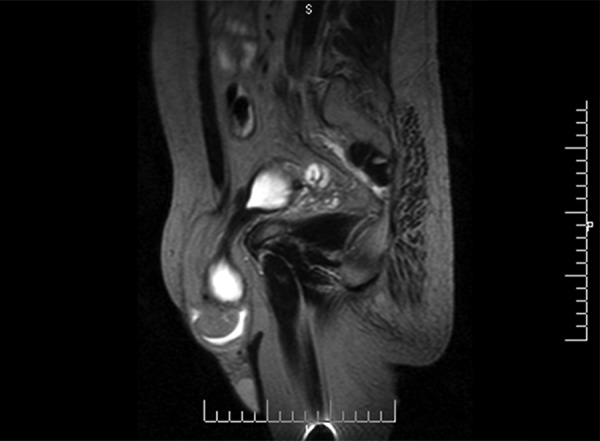
Magnetic resonance imaging (parasagittal section) showing left inguinoscrotal bladder herniation

Clinical examination showed a huge soft left inguinoscrotal swelling (Fig. 1). On per rectal examination, he had mild prostatomegaly. A diagnosis of bladder hernia was made on the basis of symptoms. Ultrasound of the left groin reported indirect inguinal hernia with bladder as content, voiding cystourethrogram, and MRI confirmed bladder herniation (Figs 2 and 3).

**Fig. 4:**
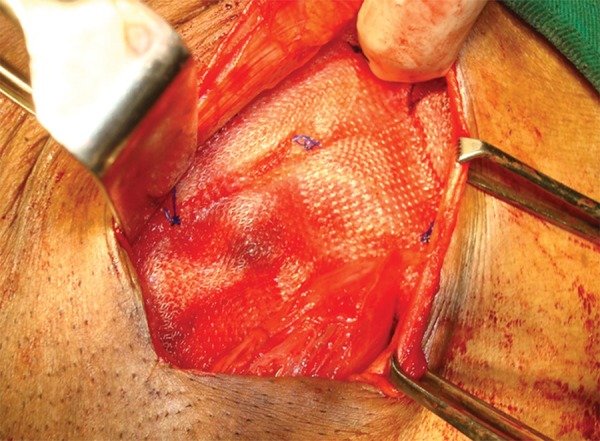
Left inguinal meshplasty after bladder was repositioned

The patient underwent inguinal hernia repair with prolene mesh after bladder catheterization. Operative finding was bladder herniation as the content in the indirect sac. Carefully, bladder was repositioned and posterior wall of the inguinal canal strengthened by prolene meshplasty (Fig. 4). The patient went home on the 8th postoperative day, and he is symptom-free on follow-up till date.

## DISCUSSION

Inguinoscrotal hernias involving the bladder are a rare entity.^1-3^ It is usually seen in patients beyond 5th decade, with a reported incidence of 10%.^4,5^ This could be explained by the fact that advancing age weakens the bladder tone and supporting structures.^6^ The preoperative diagnosis is very important to avoid iatrogenic bladder injury. The typical symptom of two-stage micturition should give a clue to the diagnosis (double micturition, manual compression of scrotal swelling to void). These patients are habituated to a two-stage micturition as the weakened detrusor contraction may be inadequate to contract and pull back the herniated portion of the bladder into the normal position in the premicturition phase; therefore, the patient adopts to and is compensated by the manual compression technique.^7,8^ Ultrasound of the swelling can differentiate bladder hernia from a hydrocele. Voiding cystourethrography demonstrates a classical dog ear herniation of the bladder.^8^ The causes, such as stricture urethra and benign prostate hypertrophy have to be considered as an underlying cause. Hernioplasty is the treatment of choice with narrowing of the internal ring.^8^

## CONCLUSION

A thorough preoperative evaluation is required to diagnose bladder hernia in all large inguinoscrotal hernias, especially in elderly patients to decrease the chances of injury at the time of surgery.
